# Mental wellbeing among people in prison in Scotland: an analysis of repeat cross-sectional surveys

**DOI:** 10.1093/pubmed/fdz106

**Published:** 2021-06-07

**Authors:** Emily J. Tweed, Xanthippi Gounari, Lesley Graham

**Affiliations:** 1 MRC/CSO Social and Public Health Sciences Unit, University of Glasgow, Glasgow G2 3AX, UK; 2 Information Services Division, NHS National Services Scotland, Edinburgh EH12 9EB, UK

**Keywords:** health inequalities, incarceration, mental health, mental wellbeing, prisons

## Abstract

**Background:**

Mental wellbeing among people in prison is poorly studied, despite featuring in many health and justice policies. We aimed to describe for the first time mental wellbeing among an unselected national prison sample.

**Methods:**

Since 2013, the Scottish Prisoner Survey—a biennial survey of people in custody in Scotland—has included the Warwick-Edinburgh mental wellbeing scale (WEMWBS), a 14-item scale with higher scores indicating greater wellbeing. We analysed data from sweeps in 2013 (*n* = 3158), 2015 (*n* = 2892) and 2017 (*n* = 2405) using Student’s *t*-test, ANOVA and multiple linear regression. We also used WEMWBS data from the Scottish Health Survey stratified by age, gender and deprivation to compare with the population at liberty.

**Results:**

Mean WEMWBS scores overall were 43.4 in 2013 (SD = 12.2), 41.8 (SD = 11.9) in 2015 and 41.2 (SD = 12.3) in 2017. Mean scores were lower among people on remand and with multiple prison episodes. Age-standardized mean scores were lower among people in prison than their peers at liberty.

**Conclusions:**

Poor mental wellbeing is an important, under-studied facet of the extreme health inequalities associated with imprisonment. These results identify that people on remand or with multiple episodes are particularly disadvantaged and provide a baseline for monitoring impacts of service or policy interventions.

## Introduction

There is growing interest in the concept of mental wellbeing, not only as an end in itself but also for its association with better physical health and longer life expectancy.^1–4^ Mental wellbeing is a related but distinct concept to mental illness: people with a diagnosis of mental illness can experience positive mental wellbeing and people may have poor mental wellbeing despite the absence of mental illness.^5^ Mental wellbeing is commonly defined as comprising both hedonic (‘feeling good’; the subjective experience of life satisfaction and happiness) and eudaimonic elements (‘doing well’; positive psychological functioning and self-realization).^6^

Though widely studied at the population level, there remain gaps in our understanding of mentalwellbeing among specific groups. In particular, although people with experience of prison are known to have very high rates of diagnosed mental illness and of mortality from related causes,^7,8^ few studies have investigated their mental wellbeing.

It is estimated that more than 10 million people are in prison worldwide at any given time; since 2000, the world prison population has grown by almost 20%.^9^ People with experience of prison are known to experience much poorer health compared to the general population,^10,11^ even after accounting for socioeconomic position and other potential confounding factors.^12^ There is growing concern about worsening mental health among people in prison in a number of countries, with prisons in England and Wales recording substantial increases in rates of self-harm and self-inflicted death in recent years.^13–15^ In Scotland, an estimated 14% of people in prison have a history of psychiatric disorder and 7.3% self-harm,^16^ while 78% test positive for illicit substances at reception.^17^

Alongside these concerns about poor health outcomes, there is a long-standing interest in the ‘health-promoting’ potential of prisons, as a setting in which positive health and wellbeing can be fostered.^18^ One manifestation of this is the strong emphasis on wellbeing in many health and justice policies. For instance, the World Health Organisation’s Trenčín Statement on prison mental health states that ‘promoting mental health and wellbeing should be central to a prison’s health care policy,’^19^ while the Scottish Government’s Vision for Justice identifies as one of its seven priorities the improvement of health and wellbeing in justice settings.^20^

However, to date, there appear to have been no large-scale studies of mental wellbeing among unselected prison populations anywhere in the world and no investigation of differences by age, gender or custodial status. As a result, there is currently a limited evidence base for understanding the mental wellbeing of people in prison and how it might be improved.

Most studies purporting to measure mental wellbeing among prison populations have in fact used instruments that measure distress or symptoms of mental illness (e.g. ^21,22^). Others have described mental health-related quality of life, which is a distinct, though related, concept.^23,24^ The only studies to date which measure mental wellbeing appear to be limited to small subgroups of people in prison, such as specific ethnic groups^25^ or those with serious mental illness.^26^

This study therefore aimed to answer the following research questions, using a repeated cross-sectional survey of people in prison in Scotland:

(i) What is the mental wellbeing of people in prison, and does it vary by demographic and custodial characteristics?
(ii) How does mental wellbeing among people in prison compare to the population at liberty?

## Methods

### Survey methodology

The Scottish Prison Service (SPS) is responsible for all 15 prisons in Scotland and undertakes the Prisoner Survey on a biennial basis in order to inform service delivery, to enable comparisons between prisons and to track progress over time.^27^

The survey consists of a self-completed paper questionnaire distributed to all people incarcerated in all prisons in Scotland. In advance of the survey, publicity materials are displayed prominently around the prison and potential participants are given a leaflet about the survey and its aims. On the day of the survey, potential participants are issued with a form by prison staff, informed of the voluntary nature of completion and offered the opportunity to ask questions. Each person completes the survey in their cell, after which sealed envelopes containing completed forms are collected by the survey team or members of staff. Translated questionnaires are provided in selected foreign languages and interpreters are provided as necessary on the day of the survey. Participants with literacy difficulties are assisted to complete the survey by cell mates or members of staff, depending on their usual means for dealing with written material.

Data on the Prisoner Survey for calendar years 2013, 2015 and 2017 were provided in anonymized form to the authors by SPS as part of an ongoing collaboration on public health intelligence in justice settings. Due to a software malfunction at SPS, age group data were not available for the 2015 sweep.

### Measurement of mental wellbeing

Since 2013, the Prisoner Survey has included the Warwick-Edinburgh Mental Wellbeing Scale (WEMWBS), in order to measure the subjective mental wellbeing of participants. WEMWBS is a self-reported measure aiming to capture both hedonic (‘feeling good’, i.e. subjective happiness and life satisfaction) and eudaimonic (‘doing well’, i.e. positive functioning and self-realization) aspects of mental wellbeing. It consists of 14 positively worded items, such as ‘I’ve been feeling optimistic about the future’ and ‘I’ve been able to make up my own mind about things’ and is scored by summing the response to each item answered on a 1 to 5 Likert scale (‘none of the time’, ‘rarely’, ‘some of the time’, often’, ‘all of the time’).^5^ The total score ranges from 14 to 70: higher scores indicate greater wellbeing.

WEMWBS has been validated in a range of population samples and, following translation, in a number of languages.^5,28^ Validation analyses in the UK have confirmed a single underlying factor (interpreted to be mental wellbeing); good internal consistency (Cronbach’s alpha of 0.89 in a student sample of 348 and 0.91 in a general population sample of 1749); the ability to distinguish between population groups consistent with other population surveys and high correlations with other tools measuring psychological wellbeing and mental health (e.g. for the Scales of Psychological Wellbeing instrument, *r* = 0.74, *P* < 0.01).^29^ Analyses of sensitivity to change suggest that a change of ±3 points or more is likely to be recognizable to an individual.^5^ It has little or nothing in the way of ceiling and floor effects and is widely used in national and local surveys and the evaluation of public mental health initiatives.^5,28^

### Population comparisons

The Scottish Health Survey (SHeS) is an annual survey of people living in private households in Scotland, which aims to provide information on a range of health indicators and determinants of health in the Scottish population. Mental wellbeing is assessed using the WEMWBS tool, as part of a paper self-completed questionnaire. To compare mental wellbeing among people in prison to that of the Scottish population at liberty, SHeS data from 2013 and 2017 were obtained from the UK Data Service (2015 was excluded from population comparisons given that data on age from the Prisoner Survey were not available for that sweep).^30^ SHeS data on WEMWBS scores for respondents aged 16 years or more were stratified by age group and gender. Though people in prison in Scotland are known to be disproportionately drawn from the most deprived areas, data on socioeconomic circumstances are not available from the Prisoner Survey.^31^ We therefore used two comparison groups to explore the relationship between imprisonment, socioeconomic circumstances and mental wellbeing: one comprising the entire SHeS sample, reflecting the socioeconomic distribution of the Scottish population, and another comprising SHeS respondents resident in the most deprived quintile of areas, using the Scottish Index of Multiple Deprivation (SIMD). For the 2013 sweep, we calculated age-standardized mean WEMWBS scores by gender for each of the two comparison groups, using the age structure of the Scottish prison population in 2013. Unfortunately, more recent data on the age structure of the prison population are not available, precluding us from undertaking this analysis for the 2017 sweep.

### Statistical analysis

Each survey sweep was analysed separately, since the anonymized nature of the data prevented identification of individuals participating in more than one sweep. Response rates were estimated by the SPS using the population of each establishment on the day of the survey minus 10%, which is the estimated proportion of people unavailable to complete the survey due to court visits, work placement, home leave, illness or other reasons (personal communication, J. Carnie; 27).

Age was categorized into three groups (16–29, 30–49 and ≥50 years), based on the SPS definition of ‘older people in prison’ (≥50 years) and the age distribution of the prison population.

Given the sample size, parametric hypothesis tests were used to investigate the relationship between WEMWBS score and demographic/custodial variables. The relationship between binary variables (custodial status and gender) with WEMWBS score was assessed using Student’s *t* tests. For categorical variables with more than two levels (age group and number of previous custodial episodes), ANOVA was used. In the absence of individual identifiers or information about repeat participation, sweeps were assumed to be independent samples: ANOVA was therefore also used to assess trends over time. Individuals with missing data for demographic or custodial variables were excluded from hypothesis testing relating to that variable. Multiple linear regression was used to investigate the relationship between WEMWBS score and the independent variables of age group, gender, remand status and previous remand or sentenced episodes. Analyses were carried out using IBM SPSS Statistics for Windows, Version 21.0 (Armonk, NY: IBM Corp).

## Results

### Sample characteristics

**Fig. 1:**
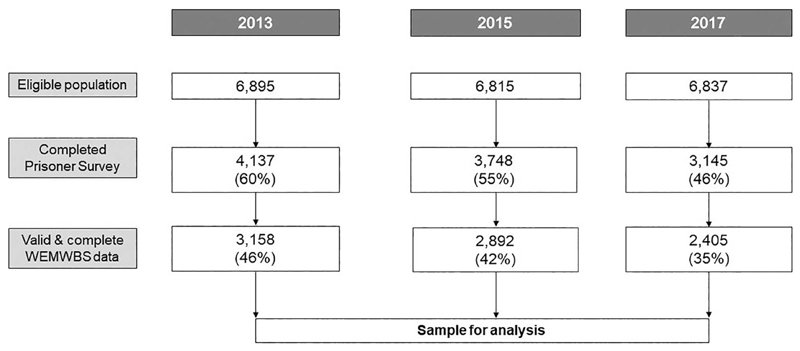
Flowchart showing derivation of sample included in analyses. Figures in brackets refer to percentage of total eligible population represented in sample.

The response rate to the Prisoner Survey as a whole was 60% (*n* = 4137/6895) in 2013, 55% (*n* = 3748/6815) in 2015 and 46% (*n* = 3145/6837) in 2017. Of those, the proportion with valid WEMWBS data that could be included in the final sample was 76% in 2013 and 2017 and 77% in 2015 (*n* = 3158 in 2013, *n* = 2892 in 2015 and *n* = 2405 in 2017), resulting in overall response rates of 46% in 2013, 42% in 2015 and 35% in 2017. The sample is shown in Fig. 1.

The demographic and custodial characteristics of the sample for each sweep are described in Table 1. The majority of the sample in each sweep were men serving a sentence; most had been in prison at least once before (whether sentenced or on remand). The characteristics of the sample were similar to those reported for the prison population as a whole, except for a slight over-representation of older people among the former (Appendix 1).

### Mental wellbeing by demographic and custodial characteristics

Table 2 describes mean WEMWBS scores for each survey sweep, according to demographic and custodial characteristics.

The overall mean WEMWBS score declined slightly in each successive sweep, with scores in both 2015 and 2017 being significantly lower than 2013 (although not significantly different to each other). In 2013, the mean score was higher among men than women (mean difference 2.1, 95% confidence interval 0.2–4.0), but no such difference by gender was observed in subsequent sweeps (2015: mean difference 1.0, 95% CI −0.85 to 2.9; 2017: mean difference −0.2, 95% CI −2.2 to 1.8). In 2013, those over 50 years of age tended to have significantly higher scores than those in younger age groups (mean difference between ≥50 years and 16–29 years −2.6, 95% CI −4.5 to −0.7; mean difference between ≥50 years and 30–49 years −3.0, 95% CI −4.7 to −1.2); this distinction by age was not apparent in the 2017 sweep.

With regard to custodial status, mean scores were consistently lower among those on remand compared to those sentenced in all three sweeps (e.g. 2017, mean difference−4.8, 95% CI −6.1 to −3.6). Mean scores were also consistently lower among those with previous prison episodes. For instance, in 2017, the mean difference between those with no previous sentenced prison episodes and those with *>*10 was −5.7 (95% CI −3.54 to −7.76). Differences by custodial characteristics were markedly greater than differences observed across other independent variables.

Results of linear regression (Appendix 2) indicate that remand status and number of previous remand episodes remained consistent predictors of WEMWBS score after adjusting for age, gender and previous sentenced episodes. However, the overall *R* ^2^ of the model was low, and collinearity between current remand status, previous remand episodes and previous sentenced episodes means that estimated coefficients may not reflect the independent effects of each variable: these results should therefore be interpreted with caution.

### Comparison to SHeS

**Fig. 2:**
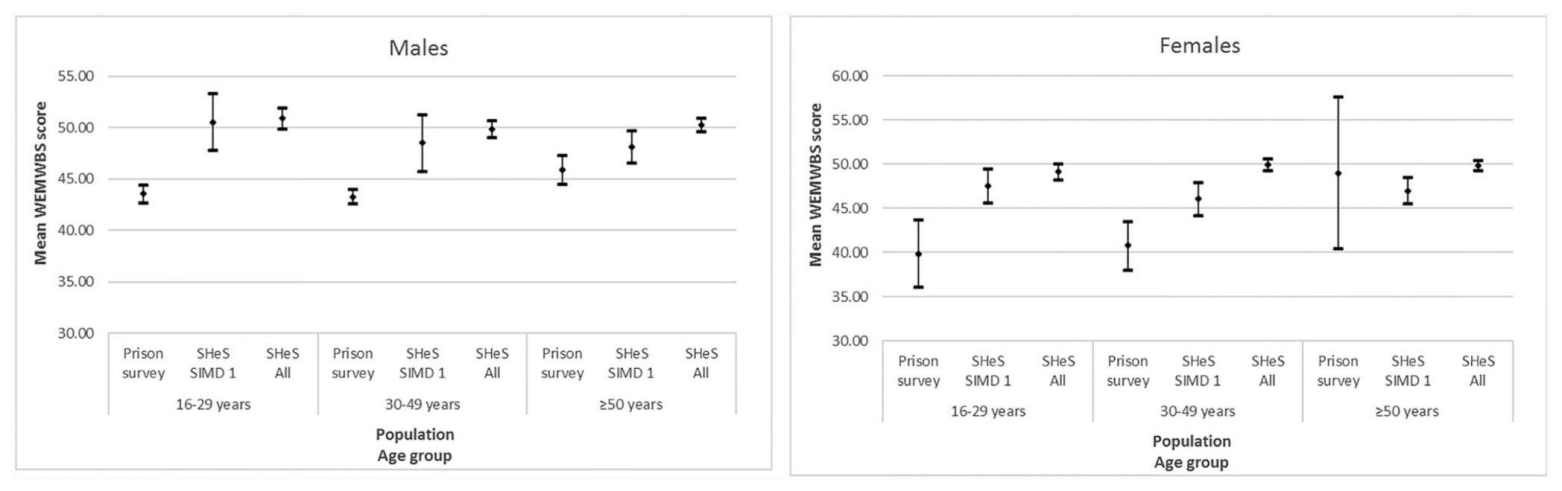
Mean WEMWBS score (and 95% confidence intervals) for respondents to the Scottish Prisoner Survey, SHeS respondents living in the 20% most deprived areas (SIMD quintile 1) and all SHeS respondents, by gender and age group (2013).

Figure 2 shows WEMWBS scores for respondents to the Prisoner Survey and respondents to SHeS, by gender, age group and socioeconomic circumstances of SHeS respondents for the year 2013. Results for 2017 were similar (Appendix 3). In all sweeps, mean WEMWBS scores were lower among people in prison than their peers of the same age group and gender living in private households, except among those aged ≥50 years. This disparity was evident whether comparing to the entire SHeS sample (reflecting the socioeconomic distribution of the Scottish population) or to those living in the 20% most deprived areas. After standardization of WEMWBS scores from the SHeS sample to the age profile of the Scottish prison population, wellbeing remained higher among people in private households for both genders in 2013 (men in prison 43.6; men in most deprived quintile 49.7; men in entire SHeS sample 50.5; women in prison 41.5; women in most deprived quintile 47.0; women in entire SHeS sample 49.7).

## Discussion

### Main findings of this study

People in prison in Scotland have poorer mental wellbeing than those at liberty, even when comparing with those living in the most deprived areas. Wellbeing was significantly lower among people on remand compared to those sentenced, with a mean difference of approximately five points across the three sweeps. There were smaller differences by year, with wellbeing being highest among those in the first compared to subsequent sweeps. Associations between higher WEMWBS and older age group or male gender were observed in 2013 but were weaker in subsequent years and did not reach conventional levels of statistical significance.

### What is already known on this topic

No previous studies have investigated mental wellbeing among small, highly selected subgroups of the prison population. Shepherd and colleagues investigated the social and emotional wellbeing of 122 Aboriginal people in prison using a bespoke culturally specific survey, which is unlikely to be transferable to Scotland.^25^ Leidenfrost et al. studied subjective wellbeing and psychological health among 43 people with serious mental illness in a New York prison, using the Schwartz Outcome Scale-10.^26^ Neither study reported comparative data or population norms or examined associations between mental wellbeing and demographic or custodial characteristics.

Previous studies examining related outcomes (such as mental health-related quality of life, self-harm or suicide) have identified that younger age, female gender and being on remand are associated with poorer mental health among people in prison.^24,32–34^

### What this study adds

To our knowledge, this is the first description internationally of mental wellbeing among an unselected, nationwide sample of people in prison.

Our sample is largely representative of the Scottish prison population as a whole and is substantially larger than the previous studies cited above. A sample of 300 is required to detect a difference of ±2 points on the WEMWBS score, suggesting that all of our subgroup analyses (except perhaps gender) are adequately powered to detect relatively subtle variations.^5^

Our findings of particularly poor mental wellbeing among people on remand are notable given concerns about its overuse and reports of poor conditions, including restricted access to support services and limited opportunities for purposeful activity.^35,36^ Efforts to improve mental wellbeing among this population may therefore be especially impactful.

Similarly, the association between multiple previous prison episodes and poor wellbeing adds to evidence that repeated short prison sentences are disruptive to family and community life, employment prospects and stable housing and are an important risk factor for mortality after release.^12, 37–39^ In this context, proposals by the Scottish Government to extend the presumption against custodial sentences of 12 months or less may have positive impacts on the health of people involved in the justice system.

In this study, age and gender were more weakly associated with mental wellbeing than custodial characteristics. However, taken alongside the existing evidence that younger people and women in prison are at greater risk of poor mental health, these findings support calls for age- and gender-sensitive approaches to improving health in justice settings.^11,40,41^

To some extent, these findings may reflect individual characteristics and pre-imprisonment experiences of the prison population, such as high rates of diagnosed mental health conditions^7^ and adverse life circumstances.^31,37^ However, they are also likely to be a bellwether of prison environment and experience. They reinforce the need for prison to be a positive, health-promoting setting and for low-threshold, timely access to psychological therapies for those who experience difficulties.^18,42^

More generally, our findings extend previous findings that imprisonment is associated with extreme health inequalities.^10,11^ We have demonstrated that poor mental wellbeing is one further manifestation of these inequalities: however, it should also be considered a potential mediator, given the evidence for its association with poorer long-term physical and mental health outcomes.^1–4, 43^

These results also demonstrate the value of routine collection of data on mental wellbeing as part of a holistic and strength-based approach to the health and welfare of people in prison. Inclusion of WEMWBS in indicator sets in justice settings would allow monitoring of trends over time and of vulnerable subgroups and the evaluation of specific service or policy interventions.

## Limitations

Coverage was incomplete and declined over successive sweeps, so there is potential non-response bias; for example associated with literacy or mental health difficulties. The validity of WEMWBS in prison populations has not been established: some questions, such as those about feeling ‘close to other people’ or ‘useful’ may be less valid measures of mental wellbeing in a prison setting. We were unable to track individuals across survey sweeps, so our analyses of time trends treated sweeps as independent. At the time of analysis, we did not have data on mental health problems, drug/alcohol use or previous trauma, all of which are relatively common among prison populations and may contribute to poor mental wellbeing.^7^ This limits our ability to draw conclusions about the causes of the observed differences in wellbeing between different subgroups within the prison population or between the prison population and those at liberty and is therefore an important area for future work.

## Conclusions

People in prison have significantly poorer mental wellbeing than their peers at liberty, even when comparing with those living in the most disadvantaged areas. In describing for the first time mental wellbeing among an unselected national prison sample, we have identified an important, under-researched facet of the extreme health inequalities experienced by people in prison. These results identify subgroups (such as those on remand and those with multiple prison episodes) that are particularly disadvantaged and provide a baseline for monitoring changes in wellbeing in response to service or policy interventions.

## Supplementary Material

Supplementary data are available at the *Journal of Public Health* online.

Supplementary information

## Figures and Tables

**Table 1:** Demographic and custodial characteristics of survey respondents with valid WEMWBS data, by survey sweep^a^

	*Survey sweep*		
	
	*2013*	*2015*	*2017*
Total sample in each sweep	3158	2892	2405
Gender			
Male	2924 (92.6%)	2495 (86.3%)	2194 (91.2%)
Female	171 (5.4%)	169 (5.8%)	152 (6.3%)
Missing	63 (2%)	228 (7.9%)	59 (2.5%)
Age group^b^			
16–29 years	808 (25.6%)	—	697 (29.0%)
30–49 years	1,222 (38.7%)	—	1,192 (49.6%)
50+ years	334 (10.6%)	—	440 (18.3%)
Missing	794 (25.1%)	—	76 (3.2%)
Custodial status			
Sentenced	2,315 (73.3%)	1,966 (68.0%)	1,848 (76.8%)
On remand	464 (14.7%)	563 (19.5%)	436 (18.1%)
Missing	379 (12%)	363 (12.6%)	121 (5.0%)
Previously in prison on remand^c^			
Never	806 (25.5%)	762 (26.3%)	697 (29.0%)
1–5 times	1,325 (42.0%)	1,193 (41.3%)	948 (39.4%)
6–10 times	334 (10.6%)	319 (11.0%)	248 (10.3%)
Over 10 times	562 (17.8%)	528 (18.3%)	357 (14.8%)
Missing	131 (4.1%)	90 (3.1%)	155 (6.4%)
Previously in prison on sentence^c^			
Never	879 (27.8%)	902 (31.2%)	798 (33.2%)
1–5 times	1,261 (39.9%)	1,081 (37.4%)	851 (35.4%)
6–10 times	310 (9.8%)	297 (10.3%)	242 (10.1%)
Over 10 times	464 (14.7%)	419 (14.5%)	296 (12.3%)
Missing	244 (7.7%)	193 (6.7%)	218 (9.1%)

a Data on the composition of the overall Scottish prison population are based on the most up-to-date official statistics published by the Scottish Government. Data on previous prison episodes are not available from this source.

b Age group data are not available for the 2015 survey sweep due to a software malfunction within the SPS.

c Data are presented for all respondents regardless of whether currently on remand or sentenced.

**Table 2:** Mean WEMWBS scores by demographic and custodial characteristics and by survey sweep

	*Survey sweep*		

	*2013*	*2015*	*2017*

	Mean (95% CI)	Mean (95% CI)	Mean (95% CI)
Total sample	43.4 (43.0–43.8)	41.8 (41.3–42.2)	41.2 (40.7–41.7)
Gender			
Male	43.6 (43.2–44.0)	41.9 (41.4–42.4)	41.2 (40.7–41.7)
Female	41.5 (39.7–43.3)	40.9 (39.1–42.6)	41.4 (39.4–43.3)
Age group^a^			
16–29 years	43.4 (42.6–44.3)	—	41.1 (40.2–42.0)
30–49 years	43.1 (42.4–43.7)	—	41.0 (40.3–41.7)
50+ years	46.0 (44.6–47.4)	—	42.0 (40.7–43.3)
Custodial status			
Sentenced	44.5 (44.0–44.9)	42.5 (42.0–43.0)	42.2 (41.7–42.8)
On remand	38.9 (37.8–40.0)	38.9 (37.9–39.8)	37.4 (36.3–38.6)
Previous episodes—remand^b^			
Never	45.0 (44.1–45.8)	43.3 (42.4–44.1)	43.5 (42.6–44.4)
1–5	44.1 (43.4–44.8)	41.8 (41.1–42.4)	41.2 (40.5–42.0)
6–10	42.7 (41.5–43.9)	42.5 (41.2–43.7)	38.7 (37.4–40.1)
10 or more	40.5 (39.6–41.5)	39.5 (38.5–40.5)	37.9 (36.7–39.1)
Previous episodes—sentenced^b^			
Never	44.9 (44.1–45.7)	43.1 (42.3–43.8)	43.5 (42.6–44.3)
1–5	44.0 (43.3–44.6)	41.7 (40.9–42.4)	40.9 (40.1–41.8)
6–10	42.8 (41.5–44.0)	41.5 (40.2–42.7)	39.4 (37.9–40.9)
10 or more	40.5 (39.4–41.5)	39.0 (37.9–40.1)	37.8 (36.5–39.1)

a Age group data are not available for the 2015 survey sweep due to a software malfunction within the SPS.

b Data are presented for all respondents regardless of whether currently on remand or sentenced.
