# Supplementary material for: Mental wellbeing among people in prison in Scotland: an analysis of repeat cross-sectional surveys
Source: J Public Health (Oxf). Author manuscript; Available in PMC 2021 Jun 11. (PMC8185554; doi:10.1093/pubmed/fdz106)

**Appendix 1. Comparison of characteristics between study sample and Scottish prison population.**

Prison statistics for Scotland have not been published by Scottish Government since the 2015 release of data for 2013/14, due to a technical issue currently under investigation. However, the data published for 2013/14 are shown below in Table A1 to provide a comparison with the survey sample analysed in this study. These data exclude individuals with missing demographic or WEMWBS data.

**Table A1. Comparison of study sample and published data on Scottish prison population: 2013**

|  | **Study sample (2013 sweep)** | | **Prison population 2013-14*** | |
| --- | --- | --- | --- | --- |
|  | **n** | **%** | **n** | **%** |
| **Male** | 2,924 | 94.5 | 7,462 | 94.5 |
| **Female** | 171 | 5.5 | 432 | 5.5 |
|  |  |  |  |  |
| **16-29 years** | 808 | 34.2 | 3,230 | 41.0 |
| **30-49 years** | 1,222 | 51.7 | 3,869 | 49.1 |
| **≥50 years** | 334 | 14.1 | 784 | 9.9 |
|  |  |  |  |  |
| **On remand** | 464 | 16.7 | 1,474 | 18.7 |
| **Sentenced** | 2,315 | 83.3 | 6,420 | 81.3 |

*Source: Scottish Government (2015). Prison statistics and population projections Scotland: 2013-14. Edinburgh, Scottish Government. Available from: <https://www.gov.scot/Topics/Statistics/Browse/Crime-Justice/PubPrisons>

Some data on the demographics of the prison population were also available from the Prisoner Survey report for 2017. These are shown below in Table A2 to provide a comparison with the survey sample. Unfortunately these data do not include age group and use ‘untried/convicted’ as categories of custodial status, rather than on remand/sentenced. These are slightly distinct as an individual may be convicted yet not yet sentenced, and hence still on remand.

**Table A2. Comparison of study sample and published data on Scottish prison population: 2017**

| **Study sample (2017 sweep)** | | | **Prison population* 2017** | | |
| --- | --- | --- | --- | --- | --- |
|  | **n (%)** | |  | **n (%)** | |
| **Male** | 2,194 (94%) | | **Male** | 7,218 (95%) | |
| **Female** | 152 (6%) | | **Female** | 378 (5%) | |
|  |  |  |  |  |  |
| **On remand** | 436 (19%) | | **Untried** | 1,360 (18%) | |
| **Sentenced** | 1,848 (81%) | | **Convicted** | 6,236 (82%) | |

*Source: Scottish Prison Service (2018). Prisoner Survey 2017. Edinburgh, Scottish Prison Service. Available from: <http://www.sps.gov.uk/Corporate/Publications/Publication-5751.aspx>

**Appendix 2. Results of multiple linear regression analyses.**

Multiple linear regression was undertaken, including all variables considered to be potentially important predictors of WEMWBS based on descriptive analyses. No significant interactions were identified: interaction terms are therefore not included in the final model described below. Note that the collinearity between current remand status, previous remand episodes, and previous sentenced episodes means that estimated coefficients may not reflect the independent effects of each variable and should therefore be interpreted with caution. The R^2^ of the regression model for each sweep was low, suggesting that the factors included are responsible for a relatively small proportion of the observed variation in WEMWBS within the sample.

|  | **Survey sweep** | | | | |
| --- | --- | --- | --- | --- | --- |
|  | **2013** | | **2015** | | **2017** |
|  | Coefficient (95% CI) | | Coefficient (95% CI) | | Coefficient (95% CI) |
| **Gender** |  | |  | |  |
| Male | Ref | | Ref | | Ref |
| Female | -3.67 (-5.95, -1.38) | | -1.14 (-3.19, 0.91) | | -0.39 (-2.48, 1.70) |
| **Age group^1^** |  | |  | |  |
| 16-29 years | Ref | | - | | Ref |
| 30-49 years | 0.29 (-0.89,1.47) | | - | | -0.08 (-1.25, 1.10) |
| 50+ years | 1.09 (-0.56, 2.73) | | - | | -0.03 (-1.57, 1.51) |
| **Custodial status** |  | |  | |  |
| Sentenced | Ref | | Ref | | Ref |
| On remand | -4.98 (-3.55, -6.40) | | -2.97 (-4.18, -1.77) | | -4.26 (-5.58, -2.95) |
| **Previous episodes – remand^2^** | |  | | |  |
| Never | Ref | | Ref | | Ref |
| 1-5 | 0.05 (-1.72, 1.82) | | -1.01 (-2.52, 0.50) | | -0.89 (-2.47, 0.70) |
| 6-10 | -1.71 (-4.26, 0.85) | | -1.34 (-3.61, 0.93) | | -2.25 (-4.71, 0.20) |
| 10 or more | -3.77 (-6.53, -1.01) | | -3.56 (-6.0,-1.10) | | -2.54 (-5.99, -0.57) |
| **Previous episodes – sentenced^2^** | | | |  |  |
| Never | Ref | | Ref | | Ref |
| 1-5 | -0.99 (-2.74, 0.75) | | -0.74 (-2.2, 0.72) | | -1.70 (-3.3, -0.15) |
| 6-10 | -0.18 (-2.72, 2.36) | | 0.54 (-1.74, 2.81) | | -2.06 (-4.48, 0.37) |
| 10 or more | -1.2 (-4.04, 1.58) | | -1.33 (-3.84, 1.18) | | -3.3 (-5.99, -0.57) |
| **R^2^ for model** | 0.05 | | 0.03 | | 0.05 |

1. Model for 2015 does not include age group as these data were not available.
2. Data are presented for all respondents regardless of whether currently on remand or sentenced.

**Appendix 3.**

**Figure 1. Mean WEMWBS score (and 95% confidence interval) for respondents to the Scottish Prisoner Survey (SPS), SHeS respondents living in the 20% most deprived areas (SIMD quintile 1), and all SHeS respondents, by gender and age group (2017).**


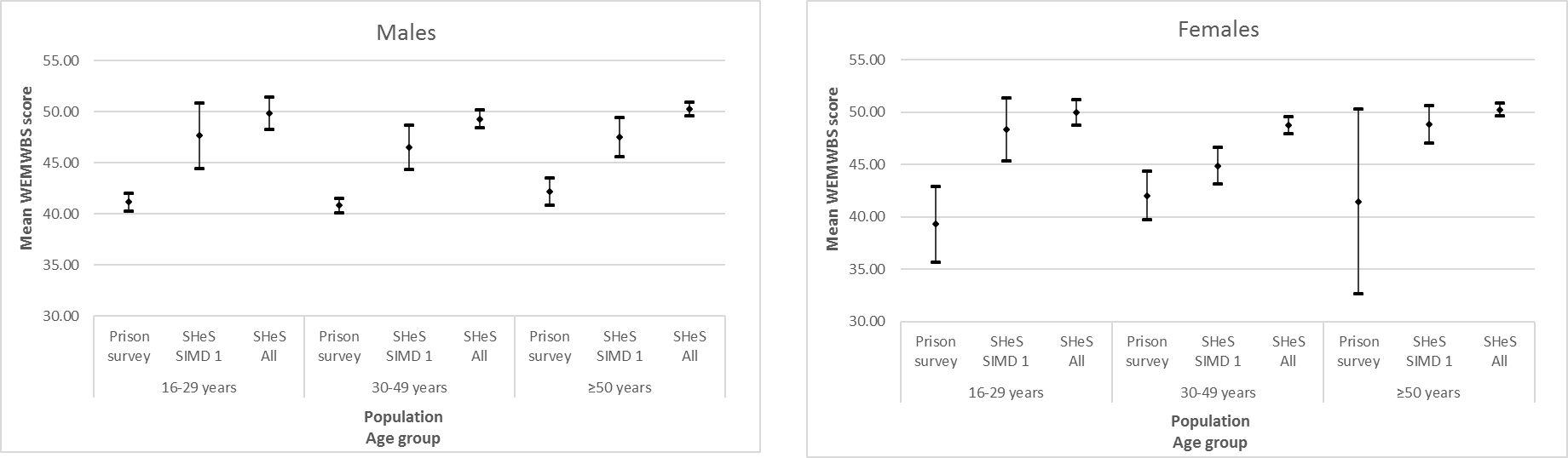

Supplement: Supplementary information [file EMS85167-supplement-Supplementary_information.docx]
